# Kinetoplastid Membrane Protein-11 as a Vaccine Candidate and a Virulence Factor in *Leishmania*

**DOI:** 10.3389/fimmu.2015.00524

**Published:** 2015-10-13

**Authors:** Sergio Coutinho Furtado de Mendonça, Léa Cysne-Finkelstein, Denise Cristina de Souza Matos

**Affiliations:** ^1^Laboratório de Imunoparasitologia, Instituto Oswaldo Cruz, Fundação Oswaldo Cruz, Rio de Janeiro, Brazil; ^2^Laboratório de Tecnologia Imunológica, Instituto de Tecnologia em Imunobiológicos, Fundação Oswaldo Cruz, Rio de Janeiro, Brazil

**Keywords:** *Leishmania*, leishmaniasis, KMP-11, virulence factor, vaccine, *Leishmania amazonensis*, *Leishmania braziliensis*

## Abstract

Kinetoplastid membrane protein-11 (KMP-11), a protein present in all kinetoplastid protozoa, is considered a potential candidate for a leishmaniasis vaccine. In *Leishmania amazonensis*, KMP-11 is expressed in promastigotes and amastigotes. In both stages, the protein was found in association with membrane structures at the cell surface, flagellar pocket, and intracellular vesicles. More importantly, its surface expression is higher in amastigotes than in promastigotes and increases during metacyclogenesis. The increased expression of KMP-11 in metacyclic promastigotes, and especially in amastigotes, indicates a role for this molecule in the parasite relationship with the mammalian host. In this connection, we have shown that addition of KMP-11 exacerbates *L. amazonensis* infection in peritoneal macrophages from BALB/c mice by increasing interleukin (IL)-10 secretion and arginase activity while reducing nitric oxide production. The doses of KMP-11, the IL-10 levels, and the intracellular amastigote loads were strongly, positively, and significantly correlated. The increase in parasite load induced by KMP-11 was inhibited by anti-KMP-11 or anti-IL-10-neutralizing antibodies, but not by isotype controls. The neutralizing antibodies, but not the isotype controls, were also able to significantly decrease the parasite load in macrophages cultured without the addition of KMP-11, demonstrating that KMP-11-induced exacerbation of the infection is not dependent on the addition of exogenous KMP-11 and that the protein naturally expressed by the parasite is able to promote it. All these data indicate that KMP-11 acts as a virulence factor in *L. amazonensis* infection.

## The Leishmaniases

Diversity is the key word for defining the leishmaniases, a group of diseases caused by the infection with parasitic protozoa of the genus *Leishmania* and transmitted by sandfly (Phlebotominae) vectors (1): diversity of parasite species, diversity of vector species, diversity of eco-epidemiological conditions involved in transmission, and diversity of clinical presentations. The leishmaniasis can be broadly classified as tegumentary (2), in which the parasitism is restricted to the integument (skin or mucosa) and visceral leishmaniasis (VL), in which internal organs like spleen, liver, bone marrow, and lymph nodes are infected. The former can be further divided into cutaneous (CL), diffuse cutaneous (DCL), and mucosal (or mucocutaneous, ML) leishmaniasis (1), according to clinical and immunopathological patterns. CL is the primary clinical form in all cases. It can be caused by all the dermotropic *Leishmania* parasites and it is, by far, the most common presentation of tegumentary leishmaniasis. ML and DCL are less frequent and more severe clinical forms, associated with distinct species and particular patterns of immune response. VL is caused by only two *Leishmania* species: *Leishmania donovani* and *Leishmania infantum* (3), but many species, belonging to two different subgenera (*Leishmania* and *Viannia*), can produce tegumentary leishmaniasis (1). While Old World CL is caused by three species, all of them of the *Leishmania* subgenus, American tegumentary leishmaniasis, so called because it encompasses CL, DCL, and ML, can be caused by various species of the *Leishmania* and the *Viannia* subgenera, the latter been exclusive of the American continent. It is currently estimated an annual incidence of 0.2–0.4 and 0.7–1.2 million cases for VL and CL cases, respectively, with a tentative estimate of 20,000–40,000 deaths per year due to VL. However, all these numbers are probably underestimated. Six countries (India, Bangladesh, Sudan, South Sudan, Ethiopia, and Brazil) account for more than 90% of global VL cases. CL has a wider geographical distribution, with the Americas, the Mediterranean basin, and western Asia being the most affected regions (4).

## Control of *Leishmania* Infection by the Mammalian Immune System

There are two major morphological stages in the life cycle of *Leishmania*: the promastigote and the amastigote. The promastigotes are the 15–20 μm long flagellated and motile forms found within the insect vectors, while the 3–5 μm long amastigotes, which lack the external flagellum, are found inside mononuclear phagocytic cells of the mammalian hosts (5).

The promastigotes undergo a differentiation process termed metacyclogenesis within the gut of the insect vector (6). Metacyclic promastigotes are the infective form for the mammalian host. They have been shown to be far more resistant to complement-mediated lysis than the procyclic promastigotes, which divide attached to the vector’s midgut epithelial cells (7). After the inoculation of the infective promastigotes by the sandfly bite, the establishment of the intracellular infection depends on a number of factors: size of inoculum (8); the phlebotomine saliva, which contains immunomodulatory molecules (9); presence of apoptotic promastigotes (10), and, especially, the ability of the parasites to survive the innate immune response of the host, which includes, among other factors, complement-mediated lysis and opsonization, phagocytosis by neutrophils and macrophages, Toll-like receptors, the NLRP3 inflammasome, and many cytokines and chemokines (11). The successful establishment of the infection results in the amastigotes dividing in phagolysosomes of macrophages, where they inhibit or subvert the killing mechanisms of these cells, making them permissive to the infection (12).

At this point, the control of the infection will depend on the adaptive immune response. Th1 CD4^+^ cells induce the activation of the parasitized macrophages through the secretion of interferon-gamma (IFN-γ) (13), with the help of other proinflammatory cytokines, such as tumor necrosis factor-alpha (14), rendering these cells capable to kill the amastigotes by producing nitric oxide (NO) and/or reactive oxygen species (ROS) (15). The generation of an effective memory T-cell response is the goal of vaccination.

## The Search for a Vaccine Against Leishmaniasis

The transmission of pathogenic *Leishmania* species is characterized by a high degree of parasite-vector specificity (16) and ~30 sandfly species are believed to be competent vectors (17). Each species has a particular ecology (18), which determines the transmission conditions and the risk factors for acquiring the disease. This diversity makes the design of control strategies extremely difficult. Moreover, the current control measures directed toward vectors and animal reservoirs have not been reliably effective (19). As a result, the geographical distribution of leishmaniasis is expanding, even to urban areas (20). On the other hand, the currently used chemotherapy regimens are toxic and expensive. Most of them have to be used parenterally for long periods (21), making adherence to therapy difficult to achieve (22). In addition, resistance to standard therapy, as pentavalent antimonials, is becoming more frequent (23). Therefore, an effective and safe vaccine could be the most comprehensive and cost-effective tool for the prevention of leishmaniasis (24).

There is no effective vaccine against any form of human leishmaniasis (24). However, during the last four decades, there have been many approaches for the development of a vaccine against leishmaniasis. Most of them stopped at the experimental level. Only a few have reached clinical trials. The majority of these were the so-called first-generation vaccine (25) candidates, composed of killed promastigotes. A major advantage of these vaccines is that they could be manufactured at low technological level and relative low cost in endemic countries (25). However, standardization of vaccines derived from cultured parasites would be impossible. Furthermore, after the various clinical trials performed with these vaccines, their efficacy has not been clearly demonstrated (26). The second-generation vaccine candidates encompass a variety of approaches: recombinant proteins, DNA, and genetically engineered organisms, such as vectored vaccines and attenuated *Leishmania*. As a rule, recombinant DNA technology is involved in their production. Their main advantages relate to safety and standardization because in this kind of vaccine, the content is precisely known. The immunization strategies mentioned above represent different modes of delivery of defined immunogens, which are, in general, parasite molecules. A number of them have been proposed as vaccine candidates, such as glycoprotein gp63, *Leishmani*a homolog of receptors for activated C kinase (LACK), kinetoplastid membrane protein-11 (KMP-11), histone H1, sterol 24-c-methyltranferase, amastigote-specific protein A2, cysteine proteinases, nucleoside hydrolase, thiol-specific antioxidant, *Leishmania major* stress-inducible protein 1, *Leishmania* elongation initiation factor, among others (27, 28). The latter three constitute a multi-subunit candidate vaccine, Leish-111F, the only recombinant candidate vaccine against leishmaniasis already tested in humans (27, 28), so far without evidence of efficacy.

## Kinetoplastid Membrane Protein-11 as a Vaccine Candidate

Kinetoplastid membrane protein-11 was discovered as a T cell-reactive contaminant (29) in preparations of lipophosphoglycan, the most abundant macromolecule on the surface of the promastigote stage of *Leishmania* spp. (30). Since then, it has been considered as a promising candidate antigen for a vaccine against leishmaniasis. It has shown an immunoprotective effect in a variety of immunization protocols (31–34).

Kinetoplastid membrane protein-11 is a protein characteristic and specific of kinetoplastid protozoa (35). The KMP-11 coding genes and their products show a remarkably high degree of sequence homology among all *Leishmania* species of both subgenera. When KMP-11 gene sequences of *L. (Viannia) panamensis*, *L. (Leishmania) infantum*, and *L. (L) donovani* were compared, a homology of more than 95% was found among them, and only three amino acid changes were found when the corresponding deduced amino acid sequences were compared (36). On the other hand, this protein shows very low homology with human proteins (37). KMP-11 has a strong antigenicity for murine (31) and human T cells (38) and is capable of stimulating both innate (39) and adaptive (38) immune responses. All these are characteristics of an ideal leishmaniasis vaccine candidate.

Another fundamental aspect for a candidate antigen for a leishmaniasis vaccine is its expression in the amastigote, the infective stage for mammals. Concerning this subject, there are interesting reports on the variability of KMP-11 expression among different species of *Leishmania*. This protein was found to be expressed at higher levels in *L. infantum* promastigotes than in amastigotes (40, 41), whereas its expression is up-regulated in amastigotes of *Leishmania amazonensis* (42) and *Leishmania mexicana* (41). It is interesting to notice that these three species belong to the *Leishmania* subgenus. To our knowledge, a similar investigation on differential expression of KMP-11 in species belonging to the *Viannia* subgenus has never been performed. Recognizing this variability is necessary for the understanding of the diversity found in the infections with different *Leishmania* species with regard to host–parasite relationship and pathogenesis. Unfortunately, this aspect has been largely neglected in leishmaniasis research. It is possible that a molecule, which plays a key role in the infection with a given *Leishmania* species would have no relevance at all for another. In this sense, it is surprising that the genomes of species causing so diverse diseases in humans like *L. major*, *L. infantum* (both from the *Leishmania* subgenus) and *Leishmania braziliensis* (*Viannia* subgenus) contain <1% species-specific genes (43). A possible explanation for this unexpected finding is that, in spite of the high similarity in their genome sequences, important differences were found between different *Leishmania* species with regard to stage-regulated gene expression (44). These differences may represent the adaptation to different vector species or the development of different strategies for survival in the mammalian host.

## Immunological Basis for Virulence Factors as Vaccines Against Leishmaniasis

During several decades, a reductionist vision has oversimplified the understanding of immunopathology of the leishmaniases. This was based in conclusions drawn from the mouse model of *L. major* infection. In this model, there is an association of resistance or susceptibility to infection with the predominance of Th1 or Th2 CD4^+^ T cell-mediated responses, respectively (45). Although this model has contributed to demonstrate the key role played by IFN-γ and Th1 cells in the control of *Leishmania* infection, it has become clear that the resistance/susceptibility to other *Leishmania* species do not fit into the so-called Th1/Th2 paradigm (46). Nevertheless, it has long guided the efforts of immunoparasitologists and vaccinologists toward the development of an anti-*Leishmania* vaccine. During this period, the Th1/Th2 paradigm was the conceptual basis for the search of potentially protective candidate antigens for a vaccine against leishmaniasis. However, this strategy eventually proved to be ineffective. *Leishmania* antigens that stimulate a Th1 immune response during the disease or even after cure were not able to induce protection when used as vaccines. On the other hand, antigens associated with disease-promoting immune responses in the early infection have been found to be highly protective if a Th1 response to them is generated by vaccination before infection (47). Probably, the best example of this is the LACK antigen which stimulates a strong Th2 response soon after infection of BALB/c mice (48) that is responsible for their extreme susceptibility to this parasite (49). However, the same antigen, when administered with adjuvants that stimulate Th1 responses (50) or as a DNA vaccine (51) protects BALB/c mice from subsequent infections with *L. major*. During coevolution, parasites have learned how to manipulate the host immune system to their own advantage by developing particular ways of antigen presentation and delivery during infection. Based on accumulating evidence, it is reasonable to believe that those evasion strategies can be overcome by defined immunization protocols using disease-promoting parasite antigens. Thus, at present, virulence factors are considered as potential drug targets and vaccine candidates for the control of leishmaniasis (52) and other infectious diseases (53).

## Kinetoplastid Membrane Protein-11 as a Virulence Factor in *Leishmania* SPP.

It has been shown that KMP-11 is a potent inducer of interleukin-10 (IL-10) production in peripheral blood mononuclear cells from patients with American CL and it is also able to inhibit the IFN-γ response of these cells to soluble *L. braziliensis* antigen extract (54, 55). IL-10 is a cytokine with anti-inflammatory properties produced by T cells, B cells, macrophages/monocytes, and keratinocytes. It can inhibit the synthesis of proinflammatory cytokines and chemokines as well as the production of NO and ROS by macrophages (56–58), restraining their ability to kill intracellular organisms (59–62).

Mukhopadhyay et al. suggested that KMP-11 may play a role in the virulence of *L. donovani* promastigotes because the loss of infective power obtained by successive sub-culturing was associated with a down-regulation of its expression (63).

Moreover, the increased expression of KMP-11 in metacyclic promastigotes, and especially in amastigotes, indicates a role for this molecule in the parasite relationship with the mammalian host, at least in members of the *L. mexicana* complex (64): *L. amazonensis* (42) and *L. mexicana* (41).

All these observations have prompted us to investigate a possible role for KMP-11 as a virulence factor in *Leishmania*. By using an *in vitro* model, we showed an exacerbating effect of KMP-11 on the infection of peritoneal macrophages from BALB/c mice with *L. amazonensis*, implicating this protein as a virulence factor for this species. This effect was higher when KMP-11 was added to the cultures 4 h after infection (and after the removal of the remaining extracellular promastigotes), as compared to simultaneously or 4 h before infection, demonstrating that the infection-promoting effect of the protein was on amastigote proliferation rather than on the internalization of promastigotes. The increase in amastigote loads was associated to an increase in IL-10 secretion and arginase activity and to an inhibition of NO production. More importantly, anti KMP-11 and anti-IL-10 antibodies were able to significantly decrease the parasite load in macrophages cultured without the addition of KMP-11, demonstrating that KMP-11-induced exacerbation of the infection is not dependent on the addition of exogenous KMP-11 and that the protein naturally expressed by the parasite is able to promote it (65).

It was recently demonstrated that poly(lactide-co-glycolide acid) nanoparticles loaded with KMP-11 induce of potent innate responses in BALB/c macrophages infected with *L. braziliensis*, promoting amastigote killing. These responses involve increased production of NO, superoxide, TNF-α and IL-6; release of CCL2/MCP-1 and CXCL1/KC; recruitment of macrophages and neutrophils *in vitro*; activation of caspase-1 and the secretion of IL-1β and IL-18 (39). Interestingly, the recombinant protein alone did not show such an effect. In contrast to our work with *L. amazonensis*, which was performed with resident peritoneal macrophages (65), thioglycolate-activated macrophages were used in this study.

The results described obtained with soluble or PLGA-coupled KMP-11 in *in vitro* infections of resident or thioglycolate-activated BALB/c peritoneal macrophages infected with *L. amazonensis* or *L. braziliensis* pose interesting questions concerning antigen delivery, macrophage activation, and differences in patterns of host–parasite relationship between different *Leishmania* species.

*Leishmania amazonensis* or *L. braziliensis* belong to different subgenera, *Leishmania* and *Viannia*, respectively (66), which are thought to have diverged 90 million years ago, when South America and Africa separated (67). Thus, New World CL is a disease caused by parasites that are quite different from each other. From the human health point of view, one of their most significant differences is the way that species from different subgenera interact with the mammalian host immunity (66).

*Leishmania amazonensis* and other members of the *L. mexicana* complex possess a remarkable ability to subvert or modulate innate and adaptive immune responses of the vertebrate host (68, 69). As a result of this, these parasites cause non-healing cutaneous lesions in most inbred strains of mice (68), although differences in susceptibility can be observed among them (69). In humans, *L. amazonensis* and *L. mexicana* are responsible for DCL, the only incurable form of human leishmaniasis, characterized by complete absence of specific type 1 response (proinflammatory, parasiticidal) to leishmanial antigens and unrestrained parasite growth (70).

*Leishmania braziliensis* and other species of the *Viannia* subgenus are not as able as the species of the *L. mexicana* complex to suppress proinflammatory and parasiticidal type 1 responses. Instead, the disease occurs in presence of an established Th1 response and IFN-γ production. Nevertheless, this response has some inhibitory effect on parasite growth. That is why parasites are less numerous in cutaneous lesions caused by *L. braziliensis* than in those produced by infection with *L. amazonensis* (66). The severe clinical form resulting from *L. braziliensis* is ML, which is associated with up-regulated Th1 responses (71). *L. braziliensis* is much less pathogenic for mice than *L. amazonensis*. Experimental infection with *L. braziliensis* can only be achieved in the BALB/c strain (72, 73).

## Conclusion and Perspectives

The presented data indicate that KMP-11 can act as a virulence factor for *L. amazonensis*, although this may not be the case for other *Leishmania* species. Future research on this subject should include the demonstration of an *in vivo* disease-exacerbating effect of KMP-11 in leishmanial infection and the evaluation of the role played by this molecule in the infection with other *Leishmania* species.

## Conflict of Interest Statement

The authors declare that the research was conducted in the absence of any commercial or financial relationships that could be construed as a potential conflict of interest.
